# BRAF V600E Mutation in Multiple Primary Malignancies: A Hairy Affair

**DOI:** 10.7759/cureus.3600

**Published:** 2018-11-16

**Authors:** Richard White, Zachary Otaibi, Rohit Rao, Gene Finley

**Affiliations:** 1 Internal Medicine, Allegheny Health Network, Pittsburgh, USA; 2 Hematology & Oncology, Allegheny Health Network, Pittsburgh, USA

**Keywords:** multiple primary malignancies, braf, braf-v600e, melanoma, hairy cell leukemia, vemurafenib

## Abstract

As the number of cancer survivors grows, so does the number of co-occurring primary malignancies and secondary malignancies. In rare cases, single driver mutations can be responsible for concomitant primary malignancies. By understanding the mechanisms that drive multiple primary malignancies (MPM), clinicians are capable of targeting molecular pathways that drive oncogenesis resulting in the successful treatment of many malignancies while also reducing the side effects of conventional chemotherapy.

Herein, we report a case of co-occurring hairy cell leukemia (HCL) and malignant melanoma in a 69-year-old male. This patient tested positive for the BRAF V600E mutation and was initiated on a single agent, vemurafenib. He, unfortunately, succumbed to his illness before completion of his planned therapy course.

This case report is intended to highlight the rare co-occurrence of BRAF-positive HCL and melanoma and to encourage driver mutation evaluation when a patient presents with MPM and the possibility of a unifying driver mutation. To the best of our knowledge, this is the first case of a co-occurring BRAF positive melanoma and HCL to be reported in a chemotherapy-naïve patient.

## Introduction

The BRAF gene encodes for a serine-threonine kinase important in cellular growth and differentiation. Activating mutations within the BRAF gene help to potentiate oncogenesis and drive many hematologic and solid organ malignancies. The most common mutation within the BRAF gene results in a substitution of glutamic acid for valine and is designated as V600E [1]. The V600E mutation accounts for 90% of BRAF mutations and has a high prevalence in metastatic melanoma, papillary thyroid cancer, colorectal cancer, and serous ovarian cancer [2-3]. Early identification has allowed for selective targeting and improvements in progression-free survival and overall survival in many cancer patients. Driver mutations are believed to be mutually exclusive; however, there are rare cases in which a single driver mutation is responsible for concurrent malignancies [4].

Herein, we report a case of co-occurring hairy cell leukemia (HCL) and malignant melanoma in a 69-year-old male. This case report is intended to highlight the rare co-occurrence of these malignancies and to add to the body of evidence that activating driver mutations can be the nidus for the development of multiple cancers in a patient simultaneously [5]. We also hope to encourage molecular evaluation in patients who present with multiple primary malignancies (MPM) when clinical suspicion arises.

## Case presentation

**Figure 1:**
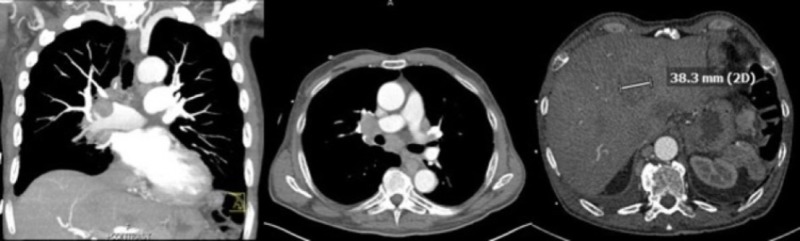
Computed tomography angiography (CTA) of the chest CTA imaging of the chest revealing a conglomerate right hilar mass involving the carina and extending inferiorly into the subcarinal, hilar, and paratracheal regions with encasement of the right main stem bronchus, bronchus intermedius, and right pulmonary artery. Also pictured is evidence of superimposed pneumonia, as well as retroesophageal and mediastinal lymphadenopathy with findings suspicious for hepatic and retrocaval metastasis.

A 69-year-old male presented with worsening cough and shortness of breath. His past medical history consisted of Parkinson's disease of two years duration refractory to medical treatment, a previous surgical splenectomy for an unknown indication, prior smoking history, and hypertension. He reported a significant decline in his health with a two to three-month history of a 40-pound weight loss and progressive dysphagia. At initial presentation, he was found to be hypoxemic and tachycardic. Computed tomography angiography (CTA) of the chest ruled out the possibility of a pulmonary embolism but revealed a conglomerate right hilar mass involving the carina and extending inferiorly into the subcarinal, right hilar, and paratracheal regions with encasement of the right mainstem bronchus, bronchus intermedius, and pulmonary artery with evidence of superimposed pneumonia. Also noted were retroesophageal and mediastinal lymphadenopathy with findings suspicious for hepatic and retrocaval metastasis (Figure 1). A complete blood count showed a white blood cell count of 1.57 K/mcL, hemoglobin of 10.0 g/dL, hematocrit of 30.2%, and a platelet count of 119 K/mcL. He was started on broad-spectrum antibiotics and admitted for treatment of pneumonia and pulmonary evaluation.

Endobronchial ultrasound with biopsy of the level 4L, 7, and 11R lymph nodes was completed. The biopsy revealed tumor cells which were positive for S100 and HMB45 and negative for CK7, CK20, P63, TTF1, and napsin-A, consistent with the diagnosis of metastatic melanoma. His pancytopenia was evaluated with a bone marrow biopsy and aspirate. The marrow showed markedly decreased trilineage hematopoiesis, a hypercellularity of 70% with extensive infiltration by B-cell lymphoma positive for CD20, DBA.44, CD25, and Annexin A and negative for CD3, CD10, CD5 and cyclin d-1; the findings were consistent with HCL. In addition, there were microscopic foci of a non-hematopoietic malignancy positive for S-100 and HMB45 consistent with malignant melanoma. It was estimated that the HCL occupied approximately 90% of the marrow cells and the melanoma cells occupied less than 5%. Whole-body bone scintigraphy was negative for bony metastatic disease. Magnetic resonance imaging of the brain was negative for intracranial metastasis. A molecular evaluation was positive for the BRAF V600E mutation in both the HCL and the melanoma.

The patient was discharged from the hospital in improved and stable condition; however, he represented to the hospital 10 days after discharge due to acute hypoxic respiratory failure secondary to new onset pulmonary edema and moderate-sized bilateral pleural effusions. The patient's respiratory status was tenuous, and he required intermittent positive pressure ventilation. Given the rapid decline in the patient's status and worsening metastatic disease, we elected to initiate him on single-agent vemurafenib at a dose of 720 mg orally twice daily. The patient received a total two doses of vemurafenib but, unfortunately, succumbed to his illness before completion of his planned therapy course.

## Discussion

MPM is a well-known but poorly understood clinical entity. One of the earliest publications regarding MPM was published in 1932 by Bugher et al. In this study, it was found that within a cancer population there was an innate susceptibility to MPM that chance alone could not explain [6]. Understanding the drivers of inherent susceptibility is complex. Furthermore, confounding bias is a limitation in many studies, as patients often have similar exposure histories and overlapping risk factors [7]. Regardless, many researchers believe that a combination of epigenetics, associated risk factors, and inherited predisposition are likely to have a role in many MPM cases [8-9].

Depending on the time interval between diagnoses, MPM can be divided into synchronous or metachronous malignancies. Synchronous malignancies are defined as primary malignancies diagnosed simultaneously or within six months following the diagnosis of the first malignancy. Metachronous malignancies are discovered six or more months apart [7]. Each primary tumor must be histologically distinct with metastatic disease eliminated as the cause of the second malignancy [10-11].

The BRAF V600E mutation is an oncogenic driver in both malignant melanoma and HCL. A study by Tiacci et al. in 2011 found that 100% of classic HCL patients harbored the BRAF V600E mutation and concluded that the BRAF V600E mutation qualifies as a disease-defining genetic event [12]. Similarly, malignant melanoma has a high prevalence of BRAF mutations and the frequency is estimated at 40 - 60% [1]. Simultaneous malignancies harboring a single oncogenic driver mutation are rare; however, if identified, it can provide clinicians with the opportunity to treat with a single agent targeted therapy. Our patient was initiated on therapy but, unfortunately, succumbed due to respiratory failure after receiving two doses of vemurafenib. Although no conclusions can be made about the effectiveness of the treatment in our patient, this case highlights the importance of early detection and underscores the need for continued research into understanding these complex and inter-related pathways of oncogenesis and optimal treatments when present.

The benefits of identifying a common driver mutation in MPM are exemplified in a case report published in 2013 by Blachly et al. [5]. They reported a case of a 67-year-old male initially diagnosed with classic HCL treated with cladribine at the time of the diagnosis, and two years later after the discovery of relapsed disease, the patient received salvage pentostatin. The patient achieved an adequate response and was managed expectantly for five years until the development of an 8 mm nodular melanoma. The patient had a wide local excision and a negative sentinel lymph node. Follow-up positron emission tomography (PET) one year later revealed a reoccurrence of melanoma at the site of excision. This patient’s recurrent melanoma tested positive for the BRAF V600E, and he underwent repeat bone marrow biopsy which was also found to be BRAF positive. The patient was treated with single-agent dabrafenib with a complete response of both tumors after six cycles [5]. A follow-up publication two years later reported that after 18-cycles of dabrafenib there had been no evidence of recurrent HCL or melanoma [13].

A similar case report documented from 2017 showed a similar scenario of a 62-year-old female with a history of HCL treated with cladribine and rituximab after relapse. Upon presentation for aphasia and forgetfulness, she was found to have three brain lesions on computed tomography. Immunohistochemistry of the lesions confirmed melanoma and a bone marrow biopsy indicated relapsed HCL. Molecular studies pointed towards a BRAF V600E mutation in both the brain melanoma and HCL. She underwent systemic, targeted therapy with dabrafenib and trametinib with a complete radiographic resolution of her brain lesions and improvement of her neurological symptoms. At one year follow-up, she showed no signs of overt disease [14].

Secondary malignancies in the setting of HCL have been the subject of an immense amount of research which has yielded mixed results. In one of the largest studies analyzing the occurrence of secondary malignancies, 3,104 HCL survivor cases registered in the National Cancer Institute’s Surveillance, Epidemiology and End Results (SEER) Program from 1973-2002 were reviewed. Their results reached statistical significance and found a 1.24-fold increase in secondary malignancies in HCL survivors relative to the general population. Hodgkin's lymphoma, non-Hodgkin's lymphoma, and thyroid cancers were reported to have the highest standardized incidence ratios (SIR). Melanoma SIR was reported at 1.40 with a total of 13 cases observed [15]. A similar study from the Memorial Sloan-Kettering Cancer Center evaluated a 267 patient cohort with an HCL diagnosis. Of these, 34 (12.7%) developed skin cancer, 11 (4.1%) developed melanoma, and 25 (9.4%) developed nonmelanoma skin cancer. The risk of developing melanoma was significantly higher in the HCL cohort (0.02%/year) than the general population. These patients had previously been treated with purine analog therapy, and the immunosuppression induced by therapy in combination with immunosuppression from HCL itself was presumed to be the aligning cause [16].

It is unclear what potentiates the occurrence of secondary malignancies in HCL patients. Many have argued that treatment with purine analogs may increase the rate of secondary malignancies, but results have been mixed. Compared to the Blachly et al. publication, the absence of prior chemotherapy in our case substantiates previous claims that treatment with purine analogs does not significantly increase the risk for secondary malignancies [17]. Others have argued that the immunosuppression associated with lymphoid neoplasms may be responsible for the development of secondary malignancies [18]. This case report adds to the body of evidence that driver mutations may, in fact, play a role in the pathogenesis of synchronous and metachronous malignancies in select cases. Although it would be difficult to discern retrospectively what percentage of 13 patients from the above study may have harbored the BRAF V600E mutation, an increased level of awareness is needed, and mutation evaluation should be encouraged on a case-by-case basis.

## Conclusions

Driver mutations are becoming an integral part of the oncologic evaluation and are important for achieving optimal treatment and high-quality cancer care. MPM is an area of increasing interest as cancer survivorship has become an important aspect in both oncologic and primary care. Histologic tissue diagnosis has been the mainstay of characterizing reoccurrences and new primary tumors in cancer survivors, but with the progress made in molecular biology and molecular characterization, histologic classification solely may no longer be adequate. We hope that this case report will encourage continued research into understanding the complex molecular pathways that can drive MPM, and when the opportunity to exploit with targeted therapy presents itself, that treatment is not delayed.
